# Vedolizumab Efficacy Is Associated With Decreased Intracolonic Dendritic Cells, Not Memory T Cells

**DOI:** 10.1093/ibd/izad224

**Published:** 2023-10-14

**Authors:** Elisa K Boden, Ramya Kongala, Duncan C Hindmarch, Donna M Shows, Julius G Juarez, James D Lord

**Affiliations:** Center for Translational Research, Benaroya Research Institute, Seattle, WA, USA; Division of Gastroenterology, Oregon Health and Science University, Portland, OR, USA; Center for Translational Research, Benaroya Research Institute, Seattle, WA, USA; Center for Translational Research, Benaroya Research Institute, Seattle, WA, USA; Center for Translational Research, Benaroya Research Institute, Seattle, WA, USA; GI Drug Discovery, Takeda Pharmaceuticals, Cambridge, MA, USA; Center for Translational Research, Benaroya Research Institute, Seattle, WA, USA; Division of Gastroenterology, Virginia Mason Medical Center, Seattle, WA, USA

**Keywords:** integrin, vedolizumab, dendritic cell, T cell

## Abstract

**Background:**

Vedolizumab, an antibody blocking integrin α4β7, is a safe and effective therapy for Crohn’s disease and ulcerative colitis. Blocking α4β7 from binding its cognate addressin MAdCAM-1 on intestinal blood vessel endothelial cells prevents T cells from migrating to the gut mucosa in animal models. However, data supporting this mechanism of action in humans is limited.

**Methods:**

We conducted a cross-sectional case-control study to evaluate the effect of vedolizumab on intestinal immune cell populations while avoiding the confounding effect of resolving inflammation on the cellularity of the colonic mucosa in treatment-responsive patients. Colon biopsies from 65 case subjects receiving vedolizumab were matched with biopsies from 65 control individuals, similar in disease type, medications, anatomic location, and inflammation. Biopsies were analyzed by flow cytometry and full messenger RNA transcriptome sequencing of sorted T cells.

**Results:**

No difference was seen between vedolizumab recipients and control individuals in the quantity of any antigen-experienced T lymphocyte subset or in the quality of the transcriptome in any experienced T cell subset. Fewer naïve colonic B and T cells were seen in vedolizumab recipients than control individuals, regardless of response. However, the most striking finding was a marked reduction in CD1c^+^ (BDCA1^+^) dendritic cells exclusively in vedolizumab-responsive patients. In blood, these dendritic cells ubiquitously express high levels of α4β7, which is rapidly downregulated upon vedolizumab exposure.

**Conclusions:**

The clinical effects of vedolizumab reveal integrin α4β7-dependent dendritic cell migration to the intestinal mucosa to be central to inflammatory bowel disease pathogenesis.

**Key Messages**

- What is already known?: Vedolizumab is an effective treatment for inflammatory bowel disease that targets integrin α4β7, and is presumed to block memory CD4 T cell migration to the intestinal mucosa.
- What is new here?: Vedolizumab use was not associated with differences in the number or gene expression of any memory T cell subset in the colon, but rather a reduction of dendritic cells, which express high levels of its target integrin α4β7.
- How can this study help patient care?: This study implicates dendritic cells in inflammatory bowel disease pathogenesis and makes them an attractive target for future therapy as well as a potential predictor and indicator of vedolizumab efficacy.

## Introduction

Crohn’s disease (CD) and ulcerative colitis (UC), collectively known as inflammatory bowel disease (IBD), are chronic inflammatory conditions of the intestine that result from a dysregulated immune response to the commensal intestinal bacterial flora. Leukocyte trafficking inhibitors have become attractive therapeutics for the treatment of IBD because of their effectiveness and favorable safety profile. Vedolizumab is a monoclonal antibody that binds to the gut-specific integrin, α4β7, and is effective for induction and maintenance of remission in moderate-to-severe CD and UC.^1,2^ Natalizumab is a similar anti-integrin but blocks all α4 integrins (α4β7 and α4β1) and thus additionally inhibits leukocyte migration to the brain. Natalizumab increases the risk of progressive multifocal leukoencephalopathy, a life-threatening, virally mediated neurologic disease. The specificity of vedolizumab for α4β7 has essentially eliminated the risk of progressive multifocal leukoencephalopathy and this drug has proved to be one of the safest biologics for the treatment of IBD.^3^ However, the exact mechanisms by which vedolizumab mitigates intestinal inflammation in IBD remains unclear.

Integrins are heterodimeric transmembrane receptors that selectively regulate adhesion and transmigration of cells through the vascular endothelium in a tissue-specific fashion. Murine models suggest that antigen presentation by dendritic cells (DCs) in the presence of retinoic acid within the gut-associated lymphoid tissue results in activation of T cells and upregulation of α4β7.^4,5^ These α4β7-expressing T cells circulate peripherally until they encounter and bind to MAdCAM-1, expressed on the gastrointestinal vascular endothelium^6^ which is upregulated during intestinal inflammation and infection.^7^ Blockade of α4β7-MAdCAM-1 interaction in murine models prevents memory lymphocyte migration to the gut-associated lymphoid tissue and intestine and reduces inflammation.^8–11^ Based on these data, α4β7 blockade via vedolizumab has been presumed to reduce inflammation through its effects on the trafficking of activated memory lymphocytes to the intestinal mucosa in humans.

To date, however, human studies have shown mixed results with respect to the effect of α4β7 blockade with vedolizumab on peripheral blood or intestinal immune cell subsets. Natalizumab, through blockade of both α4β7-MAdCAM-1 and α4β1-VCAM interaction, results in significant and persistent increases in peripheral blood leukocytes including T cells, B cells, and natural killer cells,^12,13^ suggesting sequestration of leukocytes in the blood upon treatment. In contrast to natalizumab, vedolizumab treatment does not result in increased peripheral leukocyte counts or changes in the percentage of major leukocyte subsets including CD4 or CD8 T cells, natural killer, or B cells during therapy.^1,14–16^ Vedolizumab appears to reduce expression of α4β7 on some subsets of T and B cells during therapy, but this occurs regardless of clinical response and may be related to vedolizumab-mediated receptor internalization.^14,15,17^ Thus, there are little data to support that the effects of vedolizumab are mediated by peripheral sequestration of proinflammatory lymphocytes.

The direct effect of vedolizumab on intestinal leukocyte populations has proved challenging to study. All therapies that reduce intestinal inflammation in patients with IBD markedly reduce the number of intestinal leukocytes and affect the intestinal immune composition. Thus, exploring the specific consequences of α4β7 blockade on cellular trafficking has been complicated. Several groups have reported data on the effects of vedolizumab on the intestinal immune compartment^15,16,18^ but have used a longitudinal approach, often with a comparator to anti-tumor necrosis factor (anti-TNF) therapy.

In this report, we use a unique approach to study vedolizumab-induced mucosal immune changes associated with response to therapy. A cross-sectional case-control study was designed, pairing biopsies from patients with IBD on vedolizumab therapy with control biopsies from patients not on vedolizumab. Cases and control individuals were matched 1:1 for inflammation and location of biopsies as well as patient characteristics. We show that vedolizumab does not affect the proportion of memory T lymphocytes in the colon, nor did transcriptional profiling of memory CD4, CD8, and γδ T cells reveal significant qualitative effects on these cells associated with vedolizumab use. While there were decreases in naïve T and B cell populations in the colons of patients on vedolizumab, these were not associated with response to therapy. However, CD1c^+^ conventional DCs (cDC2s), which express high levels of α4β7, were significantly reduced only in the colons of vedolizumab responders, and not in nonresponders, compared with their control individuals. Thus, the anti-inflammatory effect of vedolizumab correlates with reduced DCs, rather than with lymphocyte migration to the intestinal mucosa.

## Methods

### Study Design

We employed a cross-sectional, case-control study design for the majority of this study (cohort 1), with a longitudinal analysis of a confirmatory cohort (cohort 2). A total of 130 participants in an IBD biorepository program had donated the cryopreserved colon biopsies used for the case-control portion of this study. A total of 65 IBD subjects on vedolizumab at the time of sampling were matched 1:1 with 65 control individuals not on vedolizumab (cohort 1), none of whom were exposed to anti-TNF agents at the time of sampling. An additional 25 healthy control individuals from the biorepository program provided the cryopreserved peripheral blood mononuclear cells (PBMCs) from which data in Figure 4 were generated. Thirty additional IBD patients provided biopsies prior to starting vedolizumab and/or while on vedolizumab from which data in Figure 5 were generated (cohort 2). The study was approved by the Institutional Review Board at Benaroya Research Institute, protocol IRB07109-274. All subjects provided informed consent.

As sampling occurred within standard care, outside of a clinical trial, inflammation and response to therapy was determined by the treating physician, who was blinded to experimental results. Patients persisting on therapy with no objective serologic, fecal, endoscopic and/or radiographic evidence of inflammation and no need for systemic steroids were considered responders. Patients discontinuing therapy due to ongoing inflammation, but not to adverse events, intolerance, cost, or other psychosocial reasons, were defined as nonresponders.

### Specimens

**Figure 1.**
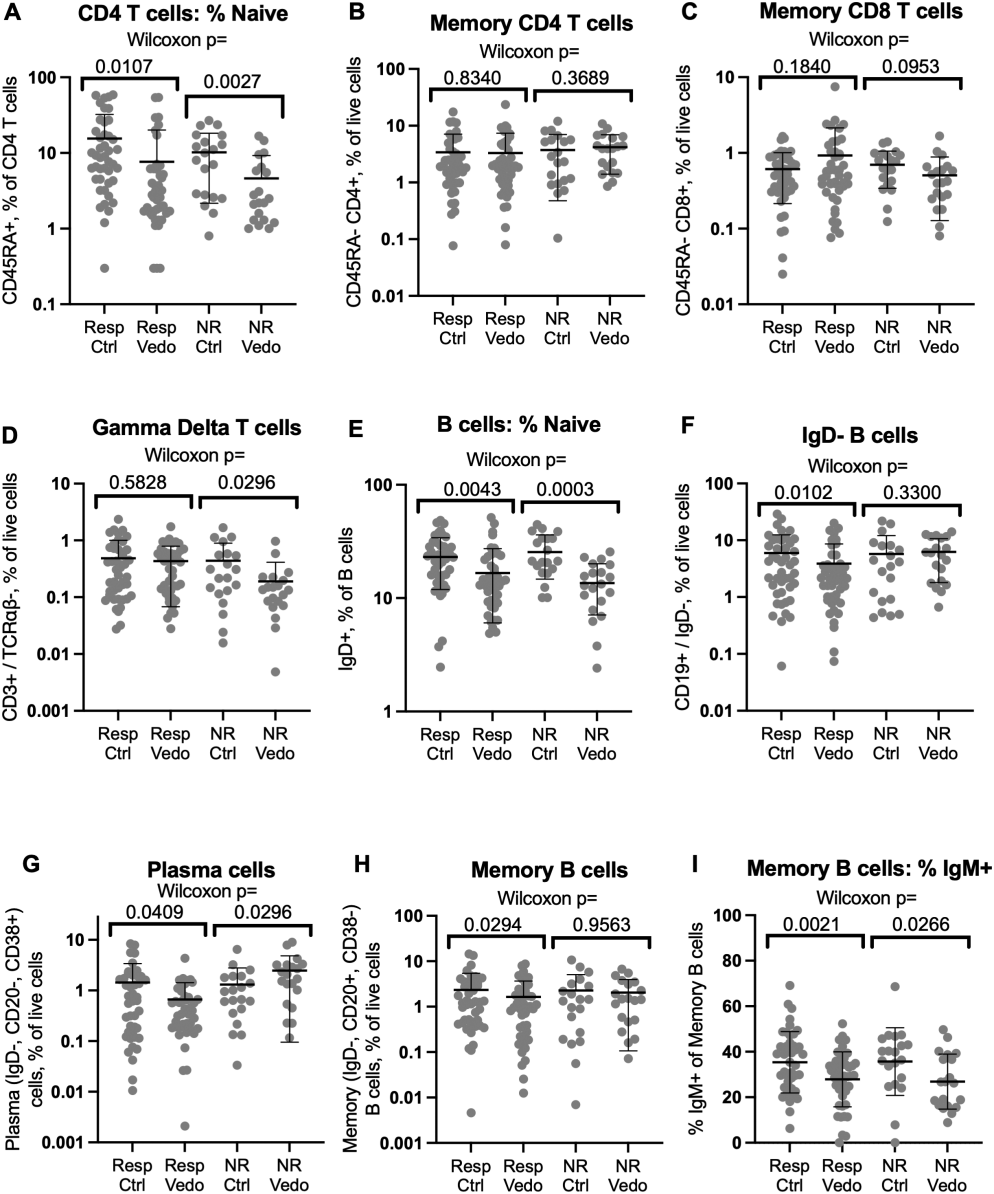
T and B cell populations in vedolizumab recipients relative to control individuals. Flow cytometry of biopsies from vedolizumab responders or nonresponders or their matched control individuals was gated on live CD3^+^, CD4^+^, or CD19^+^ lymphocytes and the percent of these cells respectively expressing CD45RA (A) or immunoglobulin D (IgD) (E) is shown on a log scale. Relative to total live cells, the percent of live CD4^+^ (B) or CD8^+^ (C) CD45RA^–^, CD3^+^, TCRαβ^+^ T cells, CD3^+^, TCRαβ^–^ gamma delta T cells (D), or CD19^+^, IgD^–^ B cells (F), and the plasma (CD20^–^, CD38^+^) (G) and memory B (CD20^+^, CD38^–^) (H) cells contained within the latter, is also shown on a log scale. The percent of memory B cells bearing surface IgM is shown in a linear scale (I). Significance of comparisons between cases and control individuals is shown by Wilcoxon signed rank test. Means and standard deviations are shown. Ctrl, control individual; NR, nonresponder; Resp, responder; Vedo, vedolizumab.

**Figure 2.**
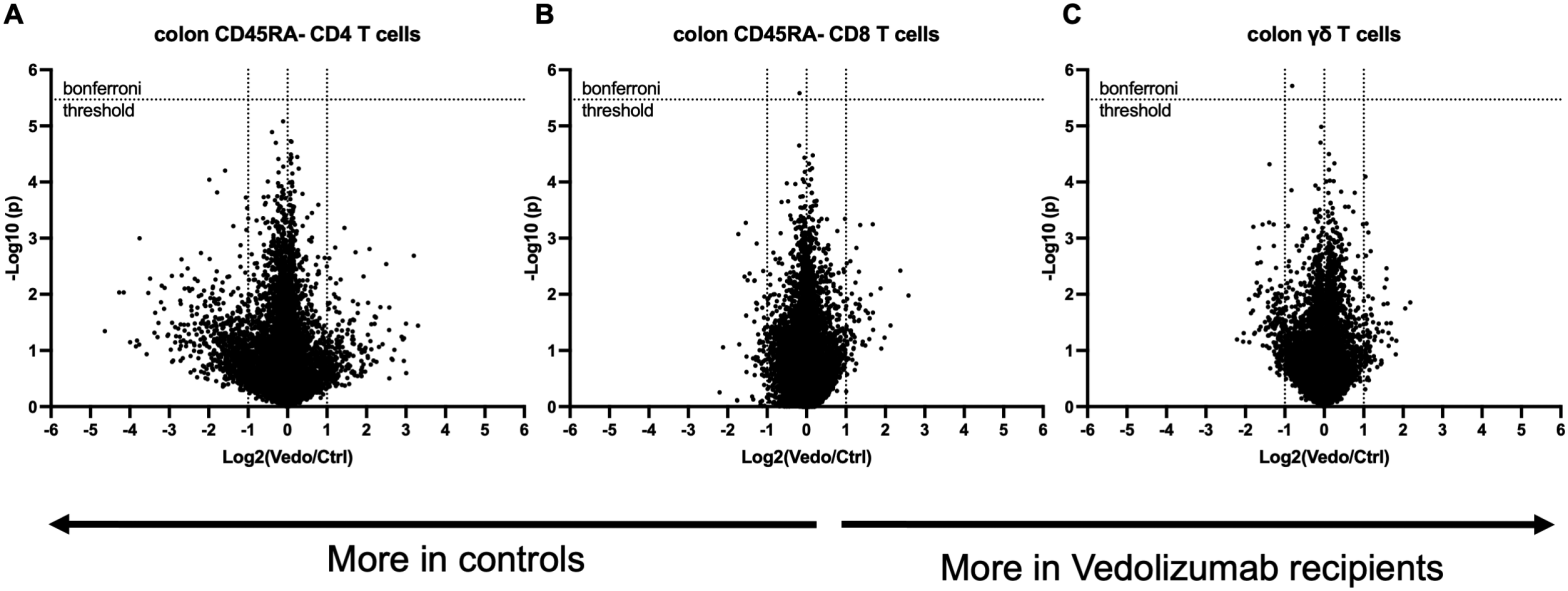
Gene expression phenotype of colonic memory T cells is not altered in vedolizumab recipients relative to control individuals. Messenger RNA was harvested and sequenced from live CD4^+^ (A) or CD8^+^ (B) CD45RA^–^, CD3^+^, TCRαβ^+^ T cells, or CD3^+^, TCRαβ^–^ gamma delta T cells (C) sorted from the colon biopsies of all study subjects. The normalized expression of every detected gene transcript in vedolizumab recipients (to the right) relative to their control individuals (to the left) is plotted on a log_2_ scale on the x-axis. The unpaired *t* test *P* value by which cases and control individuals differed for each gene is shown on a negative log_10_ scale on the y-axis. The threshold above which the latter values would need to rise to be deemed significant after Bonferroni correction for multiple comparisons is shown as a dotted line.

**Figure 3.**
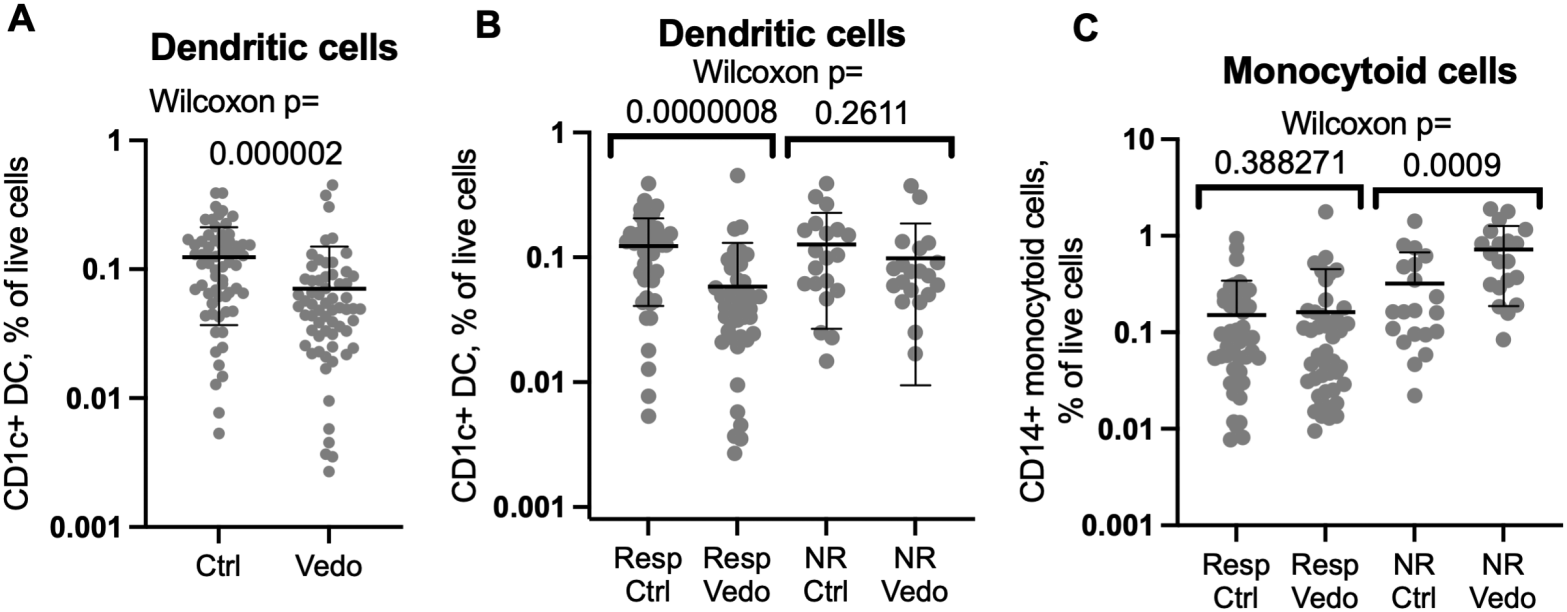
Mucosal dendritic cells are depleted in vedolizumab recipients relative to control individuals. Flow cytometry of biopsies from vedolizumab responders or nonresponders or their matched control individuals was gated on live CD1c^+^, CD14^–^ (A, B) or CD1c^–^, CD14^+^ (C) CD3^–^, CD19^–^, CD11c^+^, and HLA-DR^+^ cells. The percent of each population relative to total live cells is shown on a log scale. Significance of comparisons between cases and control individuals is shown by Wilcoxon signed rank test. Means and standard deviations are shown. Ctrl, control individual; NR, nonresponder; Resp, responder; Vedo, vedolizumab.

**Figure 4.**
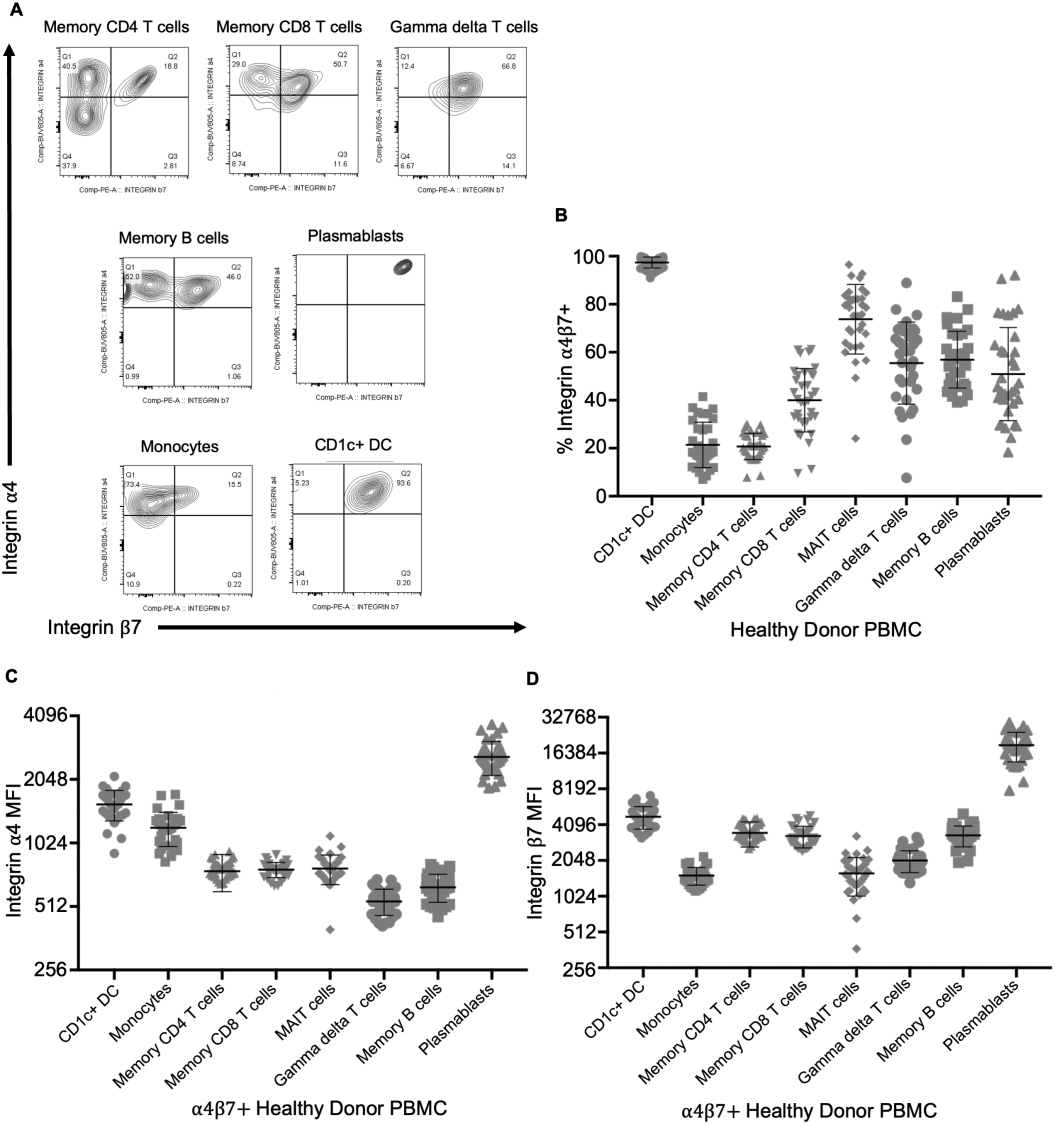
Circulating CD1c^+^ dendritic cells express more integrin α4β7 than other immune populations. Flow cytometry of peripheral blood cells from healthy donors was gated on live CD4^+^ or CD8^+^, CD45RA^–^, CD3^+^, TCRαβ^+^ memory T cells, CD3^+^, TCRαβ^–^ (presumed γδ^+^) T cells, CD19^+^, IgD^–^, CD20^+^, CD38^–^ memory B cells, CD19^+^, CD20^+^, CD20^–^, CD38^+^ plasmablasts, or CD3^–^, CD19^–^, HLA-DR^+^ cells further subdivided into CD11c^+^, CD1c^–^, CD14^+^ monocytes, CD11c^+^, or CD1c^+^, CD14^–^ dendritic cells (CD1c^+^ DC). Representative expression of integrins α4 and β7 by these cells from a single healthy donor is shown in panel A. The fraction of each population coexpressing both integrins (quadrants Q2 in panel A) (B) and the mean fluorescence intensity (MFI) of that α4β7^+^ fraction for integrin α4 (C) or β7 (D), reflecting integrin expression per positive cell, is plotted for data from the blood of 25 healthy donors. Means and standard deviations are shown. PBMC, peripheral blood mononuclear cell.

**Figure 5.**
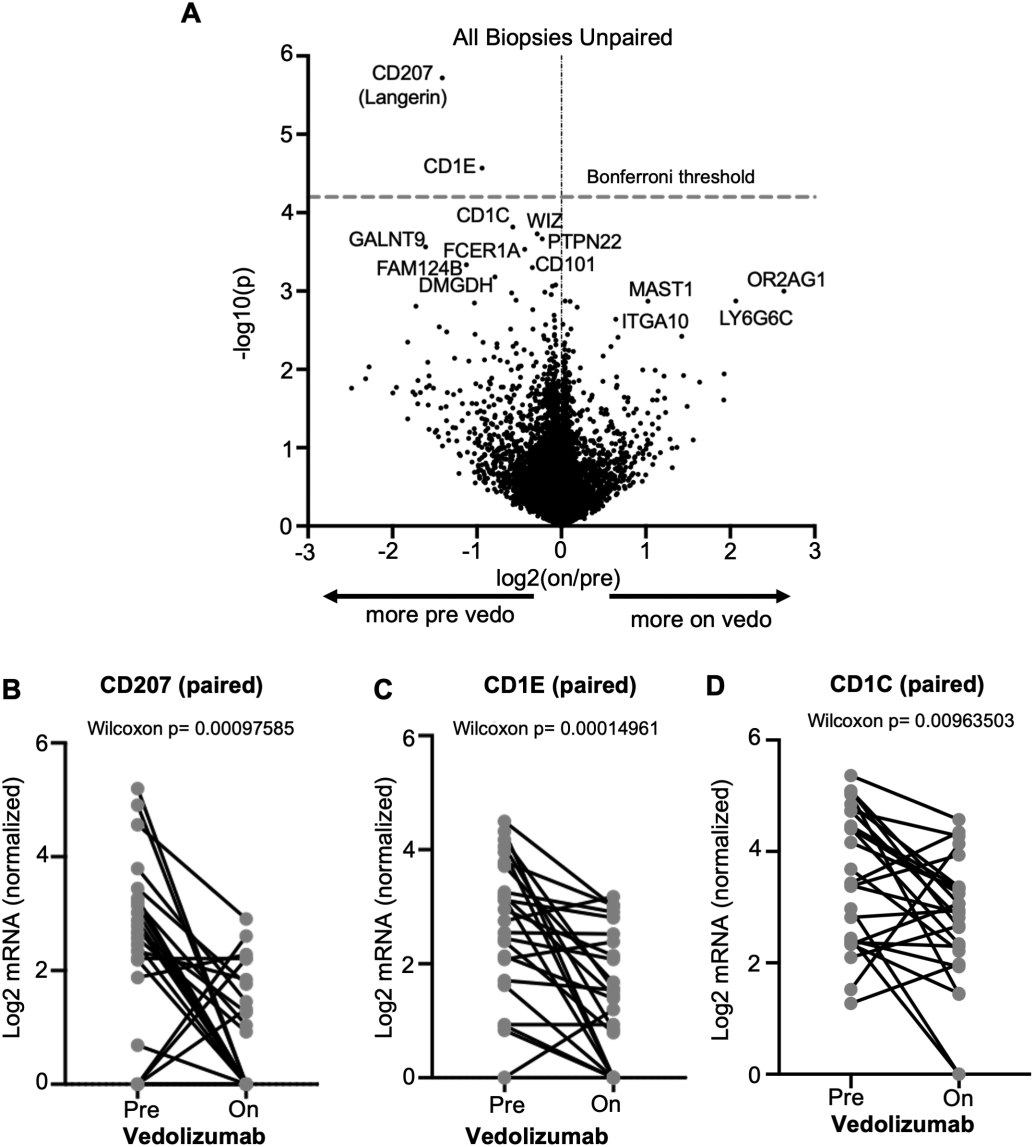
Vedolizumab decreases dendritic cell gene expression in intestinal mucosa. Messenger RNA (mRNA) was sequenced from unfractionated colonoscopic biopsies obtained from inflammatory bowel disease patients before and/or during vedolizumab therapy for inflammatory bowel disease, and the differential expression of genes based on vedolizumab exposure status is shown as a volcano plot (A). For subjects from whom biopsies were obtained both before and during vedolizumab use, paired expression of CD207 (B), CD1E (C), or CD1C (D) at the 2 time points is compared by Wilcoxon signed rank test. Vedo, vedolizumab.

Colon biopsies from which the data in Figures 1, 2, and 3 were generated were collected during standard of care colonoscopies, placed in cold fetal bovine serum, and slow-frozen in 7% dimethyl sulfoxide within an hour of each procedure. Later, thawed colon biopsies were digested in a vortex at 37 °C for up to 30 minutes in HEPES-buffered RPMI with 5% bovine calf serum containing collagenase type I and DNAse (MilliporeSigma) to liberate single cells. Digests were sedimented chilled at 200 refrigerated centrifuges for 10 minutes, and the tissues were gently homogenized through 18-gauge needles. Cells were filtered, washed, and tagged with an amine reactive dye for exclusion of highly fluorescent dead cells. Samples were washed of excess dye, then Fc receptors were blocked for at least 5 minutes using commercial cocktail and staining buffers. PBMCs from which the data in Figure 4 were generated were isolated, frozen, and later thawed using established techniques. Colon biopsies from which the data in Figure 5 were generated were collected during standard-of-care colonoscopies, placed in RNAlater buffer, and snap-frozen, to be subsequently thawed, homogenized, and extracted for RNA.

### Flow Cytometry

Fluorescent antibodies targeting cell surface lineage and secondary molecules were applied in the cold for 30 minutes, then the excess was washed away. Cells were held in 10% bovine calf serum in the cold less than 3 hours from the end of staining until capture after fluorescence-activated cell sorting.

For biopsies (Figures 1-3), fluorophore-conjugated monoclonal antibodies against CD45RA (BV610, clone HI100), CD3 (BUV395, clone UCHT1), and immunoglobulin D (IgD) (AF700, clone IAS6-2) were obtained from BD Biosciences; against CD4 (BV785, clone RPA-T4), TCRαβ (PE/Dazzle 594, clone IP26), CD19 (BV510, clone HIB19), CD20 (PE/Dazzle 594, clone 2H7), IgM (BV786, clone MHM-88), CD14 (PerCP/Cyanine 5.5, clone HCD14), CD11c (PE, clone Bu15), CD1c (APC, clone L161), HLA-DR (PE/Cyanine7, clone L243), CD38 (BV650, clone HB-7), CD161 (BV421, clone HP-3G10), CD326 (AF700, clone 9C4), and Human TruStain FcX were obtained from BioLegend; and against CD8 (APC/Cyanine7, clone RPA-T8) were obtained from eBioscience. Analysis and sorting were performed on a FACSAria Fusion Flow Cytometer (BD Biosciences) using an 85-μm nozzle. Stained T cell subsets were sorted directly into polymerase chain reaction tubes containing SmartSeq lysis buffer with double RNase inhibitor, then immediately placed on dry ice. All samples were held at −80°C until processed into RNA.

For healthy control PBMCs (Figure 4), we used antibodies against CD103 (FITC, clone Ber-ACT8), IgD (AF700, clone IA6-2), CD25 (BV786, clone M-A251), CD45RA (BV605, clone HI100), and CD3 (BUV395, clone UCHT1) obtained from BD Biosciences; against CD49d (PerCP-Cy5.5, clone 9F10), TCRVα7.2 (APC, clone 3C10), CD1c (APC, clone BDCA-1), CD4 (AF700, clone RPA-T4), CD161 (BV421, clone HP-3G10), CD19 (BV510, clone H1B19), HLA-DR (PE/Cy7, clone L243), CD11c (PE/Dazzle 594, clone Bu15), TCRαβ (PE/Dazzle 594, clone IP26), CD14 (BV785, clone M5E2), CD20 (APC-Fire 750, clone 2H7), and CD38 (BV650, clone HB-7) obtained from BioLegend; and against CD8 (APC-eF780, clone RPA-T8) and integrin β7 (PE, clone FIB504) obtained from eBioscience. Analysis was again performed on a FACSAria Fusion Flow Cytometer (BD Biosciences). Antibody panels are detailed in Supplementary Table 1.

### Integrin Internalization Assay

Thawed PBMCs from 4 healthy control donors were stained with Alexa Fluor 488 (AF488)–tagged vedolizumab, washed, and incubated at 37 °C in 10% bovine calf serum–RPMI for 0, 1.5, 6, or 24 hours. At each time point, cells were tagged with an amine reactive dye for exclusion of highly fluorescent dead cells and washed for excess dye. Then, cells were simultaneously stained with Super Brilliant Complete Staining Buffer (eBioscience) and Human TruStain FcX for at least 5 minutes to prevent nonspecific polymer interactions and block Fc receptors, respectively. Cells were then stained with antibodies against CCR2 (BUV563, clone 1D9), CCR7 (PE-Cy7, clone 3D12), CCR9 (BV480, clone C9Mab-1), CD103 (BUV395, clone Ber-ACT8), CD11b (PE-Cy5, clone ICRF44), CD127 (BUV737, clone HIL-7R-M21), CD16 (AF700, clone 3G8), CD18 (BUV496, clone 1B4/CD18), CD25 (BV786, clone M-A251), CD27 (V450, clone M-T271), CD29 (BUV615, clone MAR4), CD49d (BUV805, clone 9F10), IgD (AF700, clone IA6-2), and TCR Vδ2 (BUV563, clone B6) obtained from BD Biosciences; against CD11a (PerCP, clone TS2/4), CD123 (PerCP-Cy5.5, clone 6H6), CD56 (PerCP-Cy5.5, clone HCD56), TCRVα7.2 (APC, clone 3C10), TCRVγ9 (APC, clone B3), CD3e (BV570, clone UCHT1), CD4 (AF700, clone RPA-T4), CD161 (BV421, clone HP-3G10), CD19 (BV510, clone H1B19), HLA-DR (BV711, clone L243), CD1c (BV785, clone L161), CD11c (PE/Dazzle 594, clone Bu15), IgM (PE/Dazzle 594, clone MHM-88), TCRαβ (PE/Dazzle 594, clone IP26), and CD38 (BV650, clone HB-7) obtained from BioLegend; against CD304 (APC, clone AD5-17F6) and IgA (APC, clone IS11-8E10) obtained from Miltenyi Biotec; and against CD14 (APC-eF780, clone 61D3), CD20 (APC-eF780, clone 2H7), CD8 (APC-eF780, clone RPA-T8), or integrin β7 (PE, clone FIB504) obtained from eBioscience. In addition, a rabbit IgG anti-AF488 antibody (clone 11094; Invitrogen) capable of quenching AF488 fluorescence was applied to cells at 4 °C for 30 minutes. Cells were washed for excess antibody and stained with an Alexa Fluor 647–tagged anti-rabbit IgG antibody (goat polyclonal IgG; Invitrogen) at 4 °C for 15 minutes. Finally, cells were washed and fixed in 2% formaldehyde before being analyzed on a Cytek Aurora spectral cytometer (Cytek Biosciences). Antibody panels are detailed in Supplementary Table 1.

### RNA Sequencing

**Figure 6.**
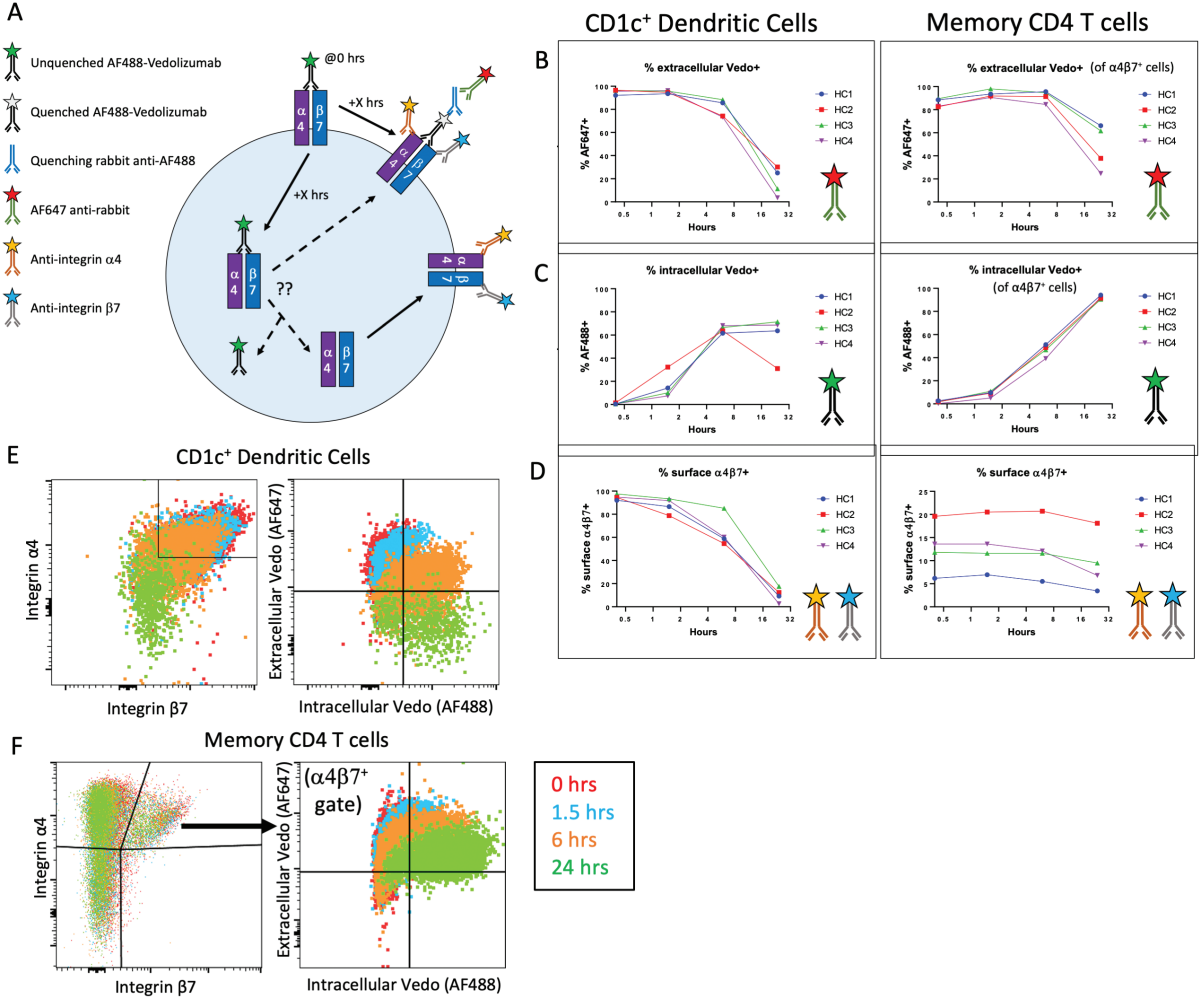
Vedolizumab causes rapid loss of surface α4β7 from dendritic cells, not T cells. (A) Cartoon depiction of reagents used to differentiate surface (B) from internalized (C) vedolizumab by using a quenching rabbit anti-Alexa Fluor 488 (AF488), while simultaneously measuring surface integrin α4 and β7 expression (D) of CD1c^+^ dendritic cells (left) or memory (CD45RA^–^) CD4 T cells (right) from the blood of 4 healthy control (HC) donors. Representative dot plots of integrin α4 and β7 (left) or extracellular (Alexa Fluor 647 [AF647]) vs intracellular (AF488) vedolizumab (right) are shown for CD1c^+^ dendritic cells (E) or memory CD4 T cells (F) from 1 donor. Unlike CD1c^+^ dendritic cells, only a fraction of memory CD4 T cells are α4β7^+^, so CD4 T cell data in panels B, C, and the right panel of panel F were gated to only show vedolizumab localization in α4β7^+^ cells.

Sorted T cells in Figure 2 (300-500 per tube) were lysed at 72 °C for 3 minutes in the presence of 3ʹ SMART CDS Primer II A, then were converted to complementary DNA using the SMART-Seq v4 Ultra Low Input RNA Kit (Takara Bio). For Figure 6, whole biopsies in RNAlater were thawed, homogenized, and converted to RNA with an RNeasy mini kit (Qiagen). Single-read sequencing of the libraries was carried out on a HiSeq 2500 sequencer (Illumina) with 58-bp reads, using SMART-seq v4 (Takara Bio) and Nextera XT kits (Illumina) with a target depth of 5 million reads. Reads were aligned to the University of California, Santa Cruz human genome assembly version 19 in Galaxy using the STAR alignment (https://www.globusgenomics.org/genomics/) tool. All libraries passed the following quality criteria: the fraction of unpaired reads examined compared with total FASTQ reads was >75%, the median coefficient of variation of coverage was <0.9, and the library had >1 million reads.

### Data Analysis and Statistics

Data were analyzed with FlowJo version 10 (BD Biosciences), Excel version 16 (Microsoft), and GraphPad Prism version 10 (GraphPad Software). For 2-way comparisons, paired comparisons between vedolizumab recipients and their 1:1 matched control individuals were made by Wilcoxon signed rank test unless continuous data was in a Gaussian distribution, in which case a paired *t* test was used. Unpaired analyses were performed by a Mann-Whitney *U* test unless they were Gaussian data, for which Student’s *t* test was used. Whole genome transcriptome profiles were normalized for total reads, log_2_ transformed, and compared between groups for each gene by paired or unpaired *t* test. Volcano plots were made by plotting the log_2_-fold change of mean normalized transcript count for each gene between comparator groups against the negative log_10_ of the *P* value from each gene’s *t* test. A threshold was set above which the latter would need to rise to meet statistical significance after Bonferroni correction for multiple comparisons, based on the total number of detectable genes compared.

## Results

### Case-Control Strategy to Reduce Influence of Inflammatory Burden Upon Immunophenotype

To differentiate the specific effects of vedolizumab on colonic immune composition from the nonspecific effect of colonic inflammation decreasing from baseline in treatment-responsive patients, we opted for a case-control, cross-sectional analysis strategy rather than a longitudinal one. This allowed for matching of biopsy samples based on the presence of inflammation, as well as other potential confounders. We retrieved cryopreserved colon biopsies from 65 patients on vedolizumab from a biorepository. As control individuals, cryopreserved colon biopsies were retrieved from another 65 IBD patients in the biorepository who were not on vedolizumab. Two adjacent biopsies were used for each donor. While most subjects were sampled at a single colon location, matched between cases and control individuals for both anatomic segment and inflammation, 1 patient from each group was sampled at 2 different but likewise matched colon locations, for a total of 66 matched specimens. Each control individual was matched to a specific vedolizumab recipient for type of IBD (CD or UC), IBD medications (other than vedolizumab), colon anatomic segment biopsied, and presence or absence of endoscopically visible inflammation at the biopsy site. Overall demographics and characteristics of vedolizumab recipients/biopsies and their control individuals (cohort 1) are shown in Table 1. Cases and control individuals were effectively matched for characteristics including age, sex, IBD type, biopsy location, and presence of endoscopic inflammation. Medications other than vedolizumab were well matched except for aminosalicylate use, which was more common in patients not on vedolizumab. This was necessary due to the paucity of uninflamed control patients taking no IBD medications. Forty-five (69%) patients had a response to vedolizumab and 20 (31%) patients did not. Responders in this cohort were more likely to have CD than nonresponders. Nonresponders had higher use of steroids (35% vs 4%) and increased endoscopic inflammation on biopsies (90% vs 24%), reflecting more active disease at the time of sampling.

**Table 1.** Patient characteristics

	Cohort 1				Cohort 2	
	Vedolizumab	Vedolizumab with clinical response	Vedolizumab without clinical response	Control	Pre-vedolizumab	Vedo
Subjects	65	45	20	65	24	23
Mean age, y	42	41	44	45	44	44
Sex						
Male	26 (40)	17 (38)	9 (45)	31 (47)	14 (29)	18 (39)
Female	39 (60)	28 (62)	11(55)	34 (53)	34 (71)	28 (61)
IBD type						
Crohn’s disease	31 (48)	24 (53)	7 (35)	32 (49)	28 (58)	28 (61)
Ulcerative colitis	28 (43)	17 (38)	11 (55)	29 (45)	18 (37)	16 (35)
Indeterminate colitis	6 (9)	4 (9)	2 (10)	4 (6)	2 (4)	2 (4)
Medications						
5-ASA	13 (20)	9 (20)	4 (20)	22 (34)	3 (12.5)	3 (13)
Steroids	9 (14)	2 (4)	7 (35)	10 (15)	9 (37.5)	8 (35)
Thiopurine	10 (15)	7 (16)	3 (15)	9 (14)	4 (17)	6 (26)
Methotrexate	3 (5)	2 (4)	1 (5)	2 (3)	0 (0)	4 (17)
Cyclosporin	1 (1.5)	0 (0)	1 (5)	0 (0)	1 (4)	0 (0)
Anti-TNF	0 (0)	0 (0)	0 (0)	0 (0)	8 (33)	1 (4)
Vedolizumab	65 (100)	45 (100)	20 (100)	0 (0)	0 (0)	100
Jak inhibitor	1 (1.5)	0 (0)	1 (5)	1 (1.5)	0 (0)	0 (0)
Biopsies	66	46	20	66	48	46
Endoscopic inflammation	29 (44)	11 (24)	18 (90)	28 (43)	32 (66)	21 (46)
Biopsy location						
Unspecified colon	0 (0)	0 (0)	0 (0)	1 (1.5)	3 (6)	2 (4)
Right colon	30 (45)	25 (54)	5 (25)	30 (45)	8 (17)	9 (20)
Transverse colon	5 (8)	4 (9)	1 (5)	4 (6)	7 (15)	9 (20)
Left colon	9 (14)	5 (11)	4 (20)	10 (15)	4 (8)	3 (6)
Rectosigmoid	22 (33)	12 (25)	10 (RS)	21 (32)	15 (31)	13 (28)
Ileum	0 (0)	0 (0)	0 (0)	0 (0)	9 (19)	10 (22)
Jejunum	0 (0)	0 (0)	0 (0)	0 (0)	2 (4)	0 (0)

Values are n or n (%), unless otherwise indicated.

Abbreviations: 5-ASA, 5-aminosalicylates; TNF, tumor necrosis factor.

Colon biopsies were thawed, collagenase-digested, and evaluated individually by flow cytometry (concatenated fluorescence-activated cell sorting plots and gating strategies in Supplementary Figure 1) by a flow cytometrist (R.K.) blinded to all clinical data. As internal validation of the endoscopist’s reported presence or absence of active inflammation at the biopsy site, the phenotype of CD326^+^ colonic epithelial cells was examined. By histology, inflammation in the colon is associated with both a higher immune to epithelial cell ratio and a marked increase in HLA-DR expression by epithelial cells.^19^ Consistent with this, epithelial cells in biopsies categorized as inflamed were, on average, about half the fraction of total live cells that were in biopsies deemed uninflamed (*P* = 6 × 10^–13^) (Supplementary Figure 2A). Even more strikingly, HLA-DR expression observed in the overwhelming majority of epithelial cells from inflamed biopsies was seldom seen in cells from uninflamed biopsies (*P* < 10^–15^) (Supplementary Figure 2B).

As biopsies were obtained during standard care, outside of a clinical trial, response to therapy was determined by the treating physician, with patients persisting indefinitely on therapy without ongoing objective inflammation or systemic steroid use deemed responders (n = 45), and patients discontinuing therapy due to inefficacy (rather than adverse events, intolerance, cost, or other psychosocial reasons) deemed nonresponders (n* *= 20). Supporting this categorization, biopsies from vedolizumab-responsive patients contained more epithelial cells (*P* = 1 × 10^–6^) (Supplementary Figure 2C) with less HLA-DR expression (*P* = 3 × 10^–7^) (Supplementary Figure 2D) than nonresponder biopsies, indicating less inflammation in the biopsies of responders than nonresponders on vedolizumab.

To evaluate how well pairing controlled for inflammation, the epithelial cell frequencies and phenotypes of cases and their control individuals were evaluated by paired analysis. Among nonresponders, a nonsignificant trend toward fewer epithelial cells (*P* = .13) more frequently expressing HLA-DR (*P* = .16) in the biopsies of vedolizumab recipients than control individuals suggests that the degree of colonic inflammation may differ between these 2 subgroups, with control individuals being slightly less inflamed (Supplementary Figure 2E, F). In contrast, epithelial cells represented a significantly larger fraction of total live cellularity in the responder subset of vedolizumab recipient biopsies than their control individuals (*P* = .006) (Supplementary Figure 2E). While this could represent marginally more inflammation in control individuals than their paired cases, HLA-DR expression in biopsies of responders and their control individuals was almost uniformly low, with no significant differences in between cases and control individuals (Supplementary Figure 2F). Thus, it is possible that epithelial cells represent a greater fraction of biopsy cellularity in vedolizumab responders than their matched control individuals if leukocytes reciprocally represent a smaller fraction of total live mucosal cells in vedolizumab recipients as a consequence of decreased trafficking to the mucosa.

### Vedolizumab Reduces Naïve, Not Memory, T Lymphocytes in the Colonic Mucosa

Although naïve lymphocytes are rare in the healthy colonic mucosa, we have previously shown that an unusually high frequency of naïve (CD45RA^+^) CD4 T cells resides in the colonic lamina propria of IBD patients, particularly in the setting of inflammation.^20,21^ A smaller fraction of CD4 T cells were naive in the colon biopsies of vedolizumab recipients than in their control individuals (Figure 1A), although by paired analyses, the difference was significant for both the nonresponder (*P* = .003) and responder (*P* = .01) cohorts. Thus, a relative paucity of naïve mucosal T lymphocytes was associated with vedolizumab use but not with efficacy. No significant associations were observed for CD45RA^+^ CD8 T cells (data not shown). However, in addition to naïve T cells, CD45RA is expressed by terminal effector/memory T cells, which represent a substantial fraction of CD8 but not of CD4 T cells, and thus may confound the evaluation of naïve CD8 cells via CD45RA.

Excluding naïve cells, we found no quantitative difference in memory αβ T cells in the colons of vedolizumab recipients relative to their respective control individuals, regardless of treatment response (Figure 1B, C). Although slightly fewer γδ T cells were observed in vedolizumab recipients within the nonresponder cohort than their respective control individuals, no such decreases were evident in the responder cohort to correlate their depletion with treatment efficacy (Figure 1D). Although integrin saturation on memory CD4 T cells was previously used for pharmacodynamic analyses of vedolizumab,^22^ no differences in memory CD4 T cell frequency (*P* > .83) (Figure 1C) or their expression of the CD161, CD38, or HLA-DR markers included in our analysis were seen between vedolizumab responders and their matched control individuals (Supplementary Figure 3).

### Vedolizumab Reduces Naïve and Experienced B Cells in the Colonic Mucosa

Similar to naïve CD4 T cells, a smaller fraction of B cells were naïve (IgD^+^) in vedolizumab exposed than matched control biopsies (Figure 1E) in both the nonresponder (*P* = .0003) and responder (*P* = .004) cohorts. IgD^–^ B cells were slightly (*P* = .01) less common in the colons of vedolizumab-responsive patients than their respective control individuals (Figure 1F), which is a difference that was reflected in both the plasma cells (*P* = .04) (Figure 1G) and memory B cells (*P* = .03) (Figure 1H) contained within this IgD^–^ population. No such decreases were seen in the nonresponder comparisons. Like naïve B cells, IgM + memory B cells were reduced in the setting of vedolizumab exposure in both responders and nonresponders (Figure 1I). However, too few plasma cells expressed IgM for comparisons, and there was no difference in IgA-expressing memory B cells or plasma cells in either vedolizumab exposed or nonexposed subjects compared with their control individuals (data not shown).

### Vedolizumab Does Not Alter Colonic T Cell Gene Expression

While collecting the above flow cytometric data, CD4, CD8, and γδ T cell populations were sorted from colon biopsies for transcriptome profiling (full gene expression data in the Gene Expression Omnibus [https://www.ncbi.nlm.nih.gov/geo] under the accession number GSE234982). Bulk messenger RNA sequencing of these populations revealed no genes that were differentially expressed by 2-fold or more between vedolizumab recipients and their control individuals with sufficient significance to withstand statistical correction for multiple comparisons (Figure 2). Excluding nonresponders and their control individuals from this analysis also did not reveal any differentially expressed genes (data not shown). Thus, despite the statistical power of over 100 subjects for each T cell type, no changes in gene expression by any of the evaluated mucosal T cell populations were significantly associated with vedolizumab use to suggest that it has a qualitative influence over T cell phenotypes in the colon.

### Decreased Colonic DCs Observed in Vedolizumab Responders

In contrast to memory lymphocytes, CD1c^+^, CD14^–^ DCs were significantly less common (*P* = 2 × 10^–6^) in the colon biopsies of vedolizumab recipients than their control individuals (Figure 3A). This difference was exclusively seen between biopsies from vedolizumab-responsive patients and their control individuals (*P* = 8 × 10^–7^). As no such difference was significant between vedolizumab recipients and their control individuals within the nonresponder cohort (*P* = .26), this observation correlates with treatment response (Figure 3B). No reduction in CD14^+^, CD1c^–^ monocytoid cells (the other major CD11c^+^, HLA-DR^+^ phagocytic mononuclear cell observed in the colon) was seen between vedolizumab responders and their control individuals (Figure 3C). Flow cytometry of blood from healthy donors (50% male, mean age 47 ± 9 years) showed that almost all the CD1c^+^ CD14^–^ DCs in circulation express integrin α4β7 (Figure 4A, B) and with a higher expression level per cell than any other circulating α4β7^+^ leukocyte, except plasmablasts (Figure 4C, D).

Efforts to sort DCs and monocytoid cells, as performed previously for T cells, resulted in RNA of insufficient quality for sequencing (data not shown). Therefore, a full transcriptome analysis was instead performed on total RNA extracted from 44 unfractionated intestinal biopsies from an independent cohort of 21 IBD patients on vedolizumab compared with 46 biopsies obtained from 23 patients prior to starting vedolizumab (16 of the 21 patients were sampled both before and during vedolizumab treatment) (full gene expression data in the Gene Expression Omnibus [https://www.ncbi.nlm.nih.gov/geo] under the accession number GSE234982). Patient characteristics can be seen in Table 1 (cohort 2). No genes were expressed more in vedolizumab recipients with sufficient significance to withstand correction for multiple comparisons, but CD207 and CD1E were both significantly enriched in the pre-vedolizumab biopsies, with CD1C nearly meeting our threshold for significance (Figure 5A). All 3 of these genes are known to be expressed by DCs, with CD1C encoding the very marker (CD1c, also called blood DC antigen 1) by which we identified and enumerated DCs by flow cytometry. Paired analyses of these 3 DC-associated transcripts in the mucosal RNA from the 16 patients who were sampled both before and during vedolizumab therapy showed that all 3 dropped significantly with treatment (Figure 5B-D), consistent with our observed exclusion of DCs from the mucosa of vedolizumab recipients, above. Thus, DCs appear to be the immune cell population most significantly affected by vedolizumab treatment and are therefore likely to be the primary therapeutic target of vedolizumab.

### DCs Rapidly Lose Surface α4β7 After Vedolizumab Exposure

To compare the direct effect of vedolizumab on DCs relative with other α4β7^+^ cells, PBMCs from 4 healthy control individuals (75% female, mean age 33 years) were exposed to fluorophore-conjugated vedolizumab. At specific time points up to 24 hours, they were exposed to a quenching antibody to differentiate intracellular from extracellular vedolizumab, while antibodies to integrin α4 and β7 chains identified the presence of surface α4 and β7 expression, regardless of vedolizumab binding (Figure 6A). We found that both CD1c^+^ DCs and α4β7^+^ CD45RA^–^ CD4 T cells rapidly internalized vedolizumab upon exposure, although more was present on the surface of T cells than DCs at 24 hours (Figure 6B, C). However, the percent of T cells with measurable α4β7 on their surface changed little over the course of the assay, suggesting that this integrin is either rapidly recycling to the surface or being produced de novo in T cells. In stark contrast, DCs went from nearly 100% to <20% surface α4β7 expression by 24 hours (Figure 6D-F). Thus, DCs rapidly lose cell surface α4β7 expression in response to vedolizumab exposure, while T cells maintain expression.

## Discussion

In this study, we have identified drug-specific effects of vedolizumab on intestinal immune populations by comparing colonic biopsies from patients with IBD receiving vedolizumab and control individuals matched for clinical characteristics and presence of mucosal inflammation. In agreement with several published longitudinal studies,^16,18^ we did not find a significant reduction in the quantity of memory T cells in the intestines of patients treated with vedolizumab. Likewise, minimal differences were found by transcriptome profiling on the quality of mucosal T cell subsets between patients with or without vedolizumab exposure. Thus, our current data do not support the hypothesis that the clinical efficacy of vedolizumab is a consequence of inhibition of intestinal trafficking in memory T lymphocytes.

We did identify modest effects of vedolizumab on the lymphocyte composition in the colon, although the majority of changes were not associated with response to therapy. Interestingly, vedolizumab resulted in changes exclusively within the naïve T cell compartment, rather than on the memory T lymphocyte populations. This is surprising, given that circulating α4β7^+^ memory T cells express significantly higher levels of α4 and β7 per cell than α4β7^+^ naïve lymphocytes and may indicate that memory lymphocyte populations can more readily access α4β7-independent trafficking pathways, such as VCAM-1-mediated recruitment through α4β1 (VLA4), which is not expressed by naïve T cells.^23^ An alternative possibility is that the majority of mucosal memory T cells are derived from tissue-resident memory cells, long-lived lymphocytes that do not recirculate and therefore do not require homing molecules to access the tissue.^24^ Indeed, a scintigraphic analysis of the in vivo homing of α4β7^+^ T cells reinfused into human donors showed that these cells do not accumulate in the intestines, but rather accumulate in the liver and spleen.^25^

Higher numbers of naïve T cells have been reported to be present in the intestine of patients with IBD compared with healthy control individuals^21,26^ and may provide a source for activated T cells within the tertiary lymphoid organs of the intestine.^27^ Reduction in naïve T cell numbers is associated with induction of remission in patients with IBD, but it is unclear whether this is a primary event that reduces the number T cells capable of becoming activated or is the effect of decreased recruitment as a consequence of reduced inflammation.^21^ Regardless, the reduction of naïve T cells was seen in both responders and nonresponders to vedolizumab and therefore is not likely to be a primary mechanism for drug efficacy.

In the B cell compartment, we also observed fewer naïve cells in the colons of patients on vedolizumab compared with their control individuals, again irrespective of responder status. Among experienced B cells, both plasma cells and memory B cells were reduced in the mucosa of vedolizumab responders, but not of nonresponders, compared with their control individuals. These findings are consistent with a small study of patients with HIV and IBD who were treated with vedolizumab and followed longitudinally.^16^ In this population, reductions in naïve and memory B cells, although not plasma cells, were observed on vedolizumab therapy. These reductions were also associated with attrition of lymphoid aggregates and were most pronounced in the terminal ileum.^16^ It should be noted that our findings in B cells were of modest statistical significance, insufficient to withstand correction for multiple comparison. However, as our cohort did not include sampling from the terminal ileum, it is possible that our study underestimated the effect of vedolizumab on B cell populations therein, which could be relevant to ileal CD.

In order to identify more subtle or qualitative changes in gene expression that might be present within the memory T cell compartment of vedolizumab-treated subjects, our group sorted CD4^+^, CD8^+^ memory T cells, and γδ T cells from subjects and their control individuals. RNA sequencing did not identify significant patterns of gene up- or downregulation in these populations associated with α4β7 blockade. This is consistent with the recent publication of RNA sequencing data from unfractionated sigmoid biopsies in vedolizumab-treated patients that primarily demonstrated changes in genes involved in innate immune pathways (pattern recognition receptors, chemokines, innate effector molecules) associated with vedolizumab efficacy.^18^ Unlike T cells, innate cells did not survive tissue homogenization and sorting with RNA of sufficient stability for us to corroborate these finding in purified populations. However, transcriptome profiling of RNA from intact mucosal biopsies did reveal that vedolizumab exposure resulted in a significant decrease in CD207 and CD1E messenger RNA transcripts, which are associated with DCs, rather than with lymphocytes or other nonmyeloid cells.

By flow cytometry, our primary finding associated with response to vedolizumab was indeed a reduction in colonic conventional CD1c^+^CD14^–^ DCs. Human DCs are characterized broadly by a lack of lineage markers (CD3, CD19/20, CD14, and CD56) and high expression of major histocompatibility complex class 2 (HLA-DR). DCs can be further classified into 4 major subsets defined by expression of cell surface markers, transcriptional profile and function: CD141^+^ conventional DCs (cDC1s), cDC2s, CD11c^–^ plasmacytoid DCs, and CD14^+^ monocyte–derived DCs. The cDC2s are highly effective at sampling for antigen and are potent activators of both CD4 and CD8 T cells.^28^ However, cDC2s are heterogeneous with some subsets secreting more proinflammatory cytokines, others capable of inducing T helper 1 responses, T helper 2 responses, T helper 17 responses, and regulatory T cell differentiation.^29,30^ Murine models have demonstrated a role for DCs in both the propagation and amelioration of intestinal inflammation,^31,32^ although translation of findings from animal studies to humans has been problematic due to a lack of shared phenotypic markers for DCs across species. The role of specific intestinal DC populations in the initiation and perpetuation of inflammation in human IBD therefore remains largely unknown.

Conventional CD1c^+^ cDC2s express very high levels of both integrins α4 and β7, with almost all of these cells staining positive for the heterodimer of the 2. In fact, per-cell expression of each integrin chain on cDC2s was higher than on all α4β7^+^ subsets of T cells, including memory T cells. Murine studies have demonstrated that migration of conventional DC precursors to the intestine requires the integrin β7 and the addressin MAdCAM-1.^33^ Thus, our findings that vedolizumab was associated with reduced mucosal cDC2s in responders, but not memory T lymphocytes, suggests that α4β7 blockade by vedolizumab reduces inflammation specifically through its ability to block the intestinal migration of proinflammatory cDC2 precursors.

A recent longitudinal study of the effect of vedolizumab on intestinal immune cell distribution also suggested vedolizumab-induced changes in the innate immune system with alterations in M1/M2 macrophage ratio, but not specifically cDC2s, associated with response to therapy.^18^ This study did not use flow cytometric analysis, but rather used CIBERSORT technology that estimates the relative abundance of certain cell types by deconvoluting bulk tissue transcriptome data with the transcriptomes of purified cellular populations isolated from peripheral blood.^18^ This may account for discrepancies with our findings if the transcriptomes of specific innate cell subsets differ too much between circulation and mucosa for CIBERSORT to accurately categorize them in tissue. Nevertheless, these data support a minimal mechanistic role for vedolizumab through blocking trafficking of memory lymphocytes, and instead directs our attention to the effects of vedolizumab on innate immune populations, and particularly mononuclear phagocytes.

Pharmacodynamic studies on vedolizumab have shown complete and durable saturation of integrin α4β7 on memory T lymphocyte populations lasting more than 100 days.^34^ However, clinical studies have shown a positive exposure response to vedolizumab with higher trough concentrations early in treatment associated with an increased probability of deep remission.^35^ Our group, and others, have shown near complete integrin receptor saturation on circulating memory T cells even at trough drug levels associated with lower rates of response.^14,36^ If inhibition of DC migration to the intestines is a primary mechanism for vedolizumab effect, it would follow that DC α4β7 saturation with vedolizumab may be a better predictor of response. Given the relatively short lifespan of DCs compared with memory T cells,^37^ it is possible that low trough levels of vedolizumab have a greater effect on receptor saturation in DCs than the T cell population. Furthermore, we now show that vedolizumab causes a rapid and nearly complete loss of α4β7 from the surface of DCs, but not of T cells, which would further selectively impair DC recruitment through intestinal MAdCAM-1. Whether DC-focused diagnostics could refine predictive biomarkers or therapeutic drug monitoring for vedolizumab recipients would be an important question for future clinical research.

The strengths of our study include the large number of subjects and the case-control design in which biopsies from IBD patients on vedolizumab were matched for inflammation and clinical characteristics with control individuals. This reduces confounding related to nonspecific immunologic changes that result from resolution of inflammation in responders. Other studies have tried to overcome this issue by comparing immunologic changes in subjects treated with vedolizumab with those receiving infliximab. However, this may be problematic, as infliximab has been reported to induce apoptosis in intestinal immune cells by signaling through membrane-bound tumor necrosis factor (TNF) expressed on lymphocytes as well as on macrophages.^38,39^ In addition, anti-TNFs have been shown to decrease the expression of the adhesion molecules ICAM-1^40^ and MAdCAM-1^41^ and thus may have their own independent effects on cellular trafficking. As anti-TNF agents may therefore not provide an ideal comparator to understand the effects of vedolizumab on specific immune cell populations, none of the subjects in our primary analysis were on anti-TNF or any biologic pharmaceuticals other than vedolizumab, as specified.

Limitations of our study are related primarily to its cross-sectional nature in that subjects were not able to serve as their own control individuals, and samples were taken from diverse rather than prescribed time points during therapy. Our study also did not include significant sampling of the ileum, which recent studies suggest may have a distinct immunologic profile from the colon,^42^ including effects on B cell composition.^16^ In addition, because of the limited material in the biopsy specimens for flow cytometry, a finite number of cell surface markers could be investigated for each patient, precluding more detailed mucosal immunophenotyping. In addition, as the assessment for inflammation on endoscopic biopsies was binary (inflamed or uninflamed), there may have been some unintended differences in the degree of inflammation in biopsies between cases and their control individuals. Last, our control group contained more patients on 5-aminosalicylates than our vedolizumab-treated group. While it is unlikely that 5-aminosalicylate therapy contributed to cellular changes in the mucosal immune system, this remains a possibility.

## Conclusions

Our study underscores that vedolizumab blockade of α4β7 has little effect on memory T populations in the intestines. While we found modest reductions in experienced B cell populations associated with Vedolizumab response, our most striking finding was a marked reduction in colonic CD1c^+^ DCs. These cells express high levels of α4β7, which are rapidly internalized upon vedolizumab exposure and should be investigated further both with respect to their role in the pathogenesis of IBD and as a predictive biomarker for therapeutic drug monitoring.

## Supplementary Material

izad224_suppl_Supplementary_Figure_S1AB

izad224_suppl_Supplementary_Figure_S1CD

izad224_suppl_Supplementary_Figure_S2

izad224_suppl_Supplementary_Figure_S3

izad224_suppl_Supplementary_Table_S1

izad224_suppl_Supplementary_Figure_Legends
