# Supplementary figures and images for: Vedolizumab Efficacy Is Associated With Decreased Intracolonic Dendritic Cells, Not Memory T Cells
Source: Inflamm Bowel Dis. 2023 Oct 14;30(5):704–17. doi: 10.1093/ibd/izad224 (PMC11063563; doi:10.1093/ibd/izad224)

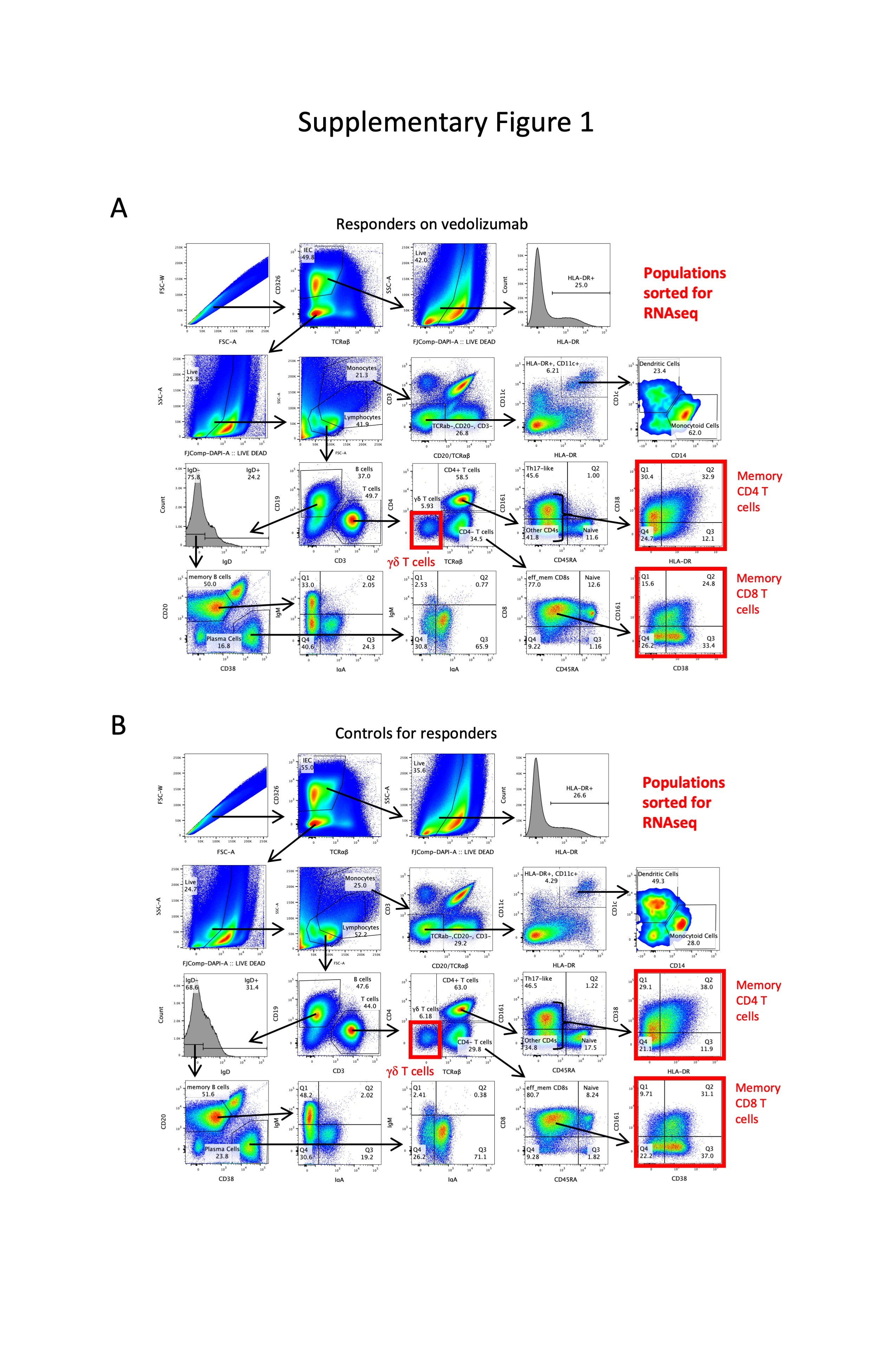

Supplement: izad224_suppl_Supplementary_Figure_S1AB [file izad224_suppl_supplementary_figure_s1ab.jpeg]

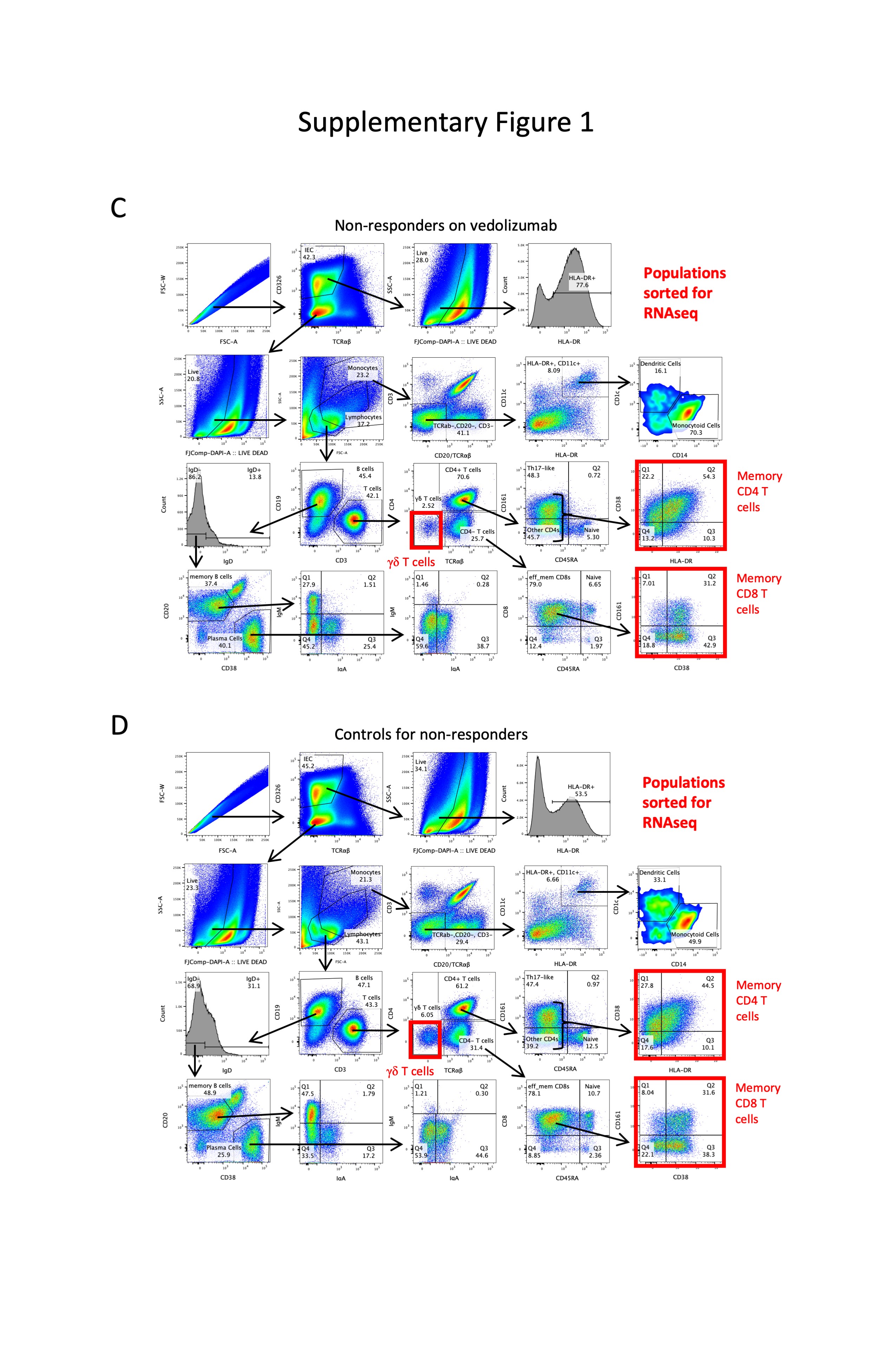

Supplement: izad224_suppl_Supplementary_Figure_S1CD [file izad224_suppl_supplementary_figure_s1cd.jpeg]

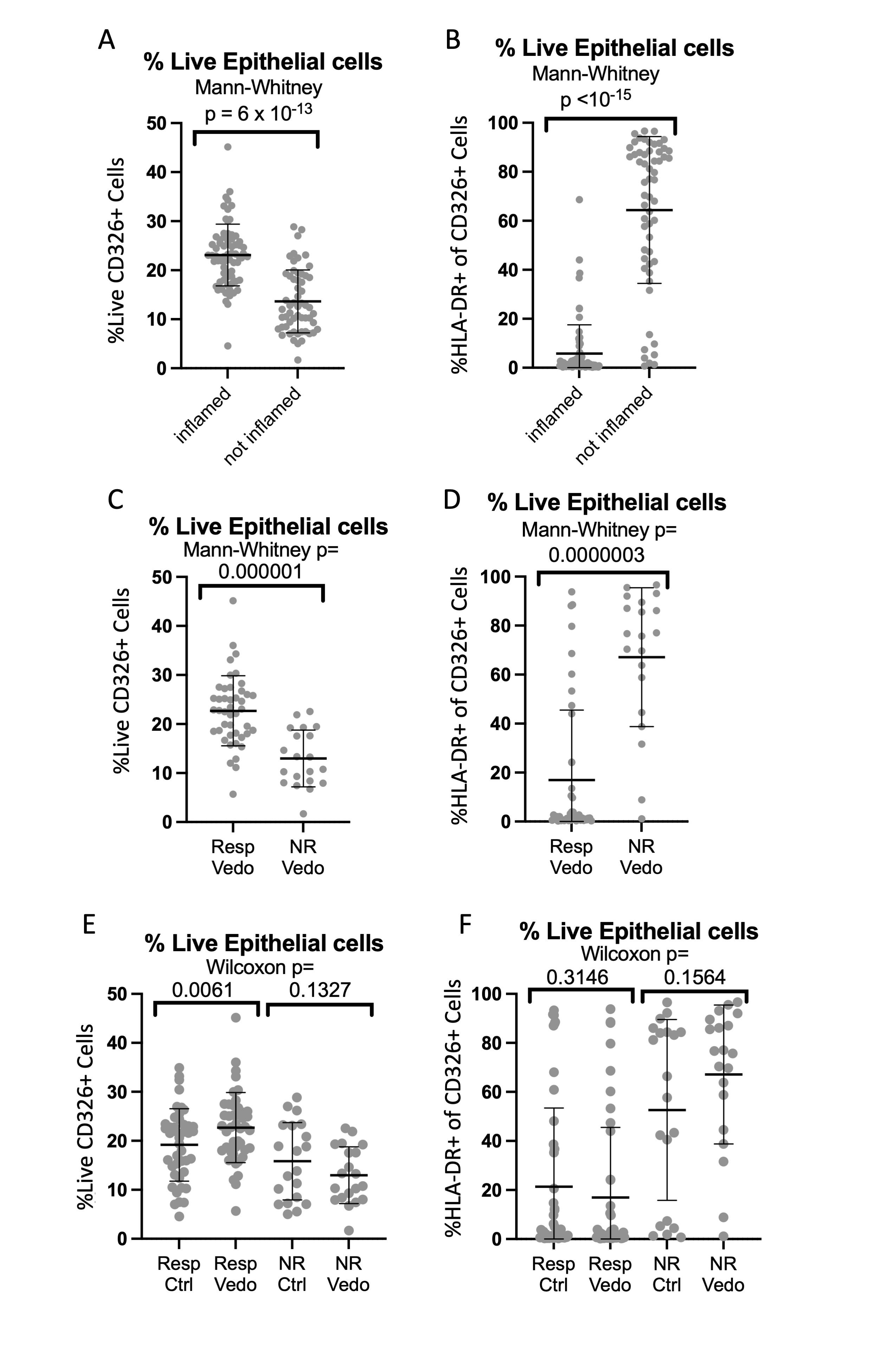

Supplement: izad224_suppl_Supplementary_Figure_S2 [file izad224_suppl_supplementary_figure_s2.jpeg]

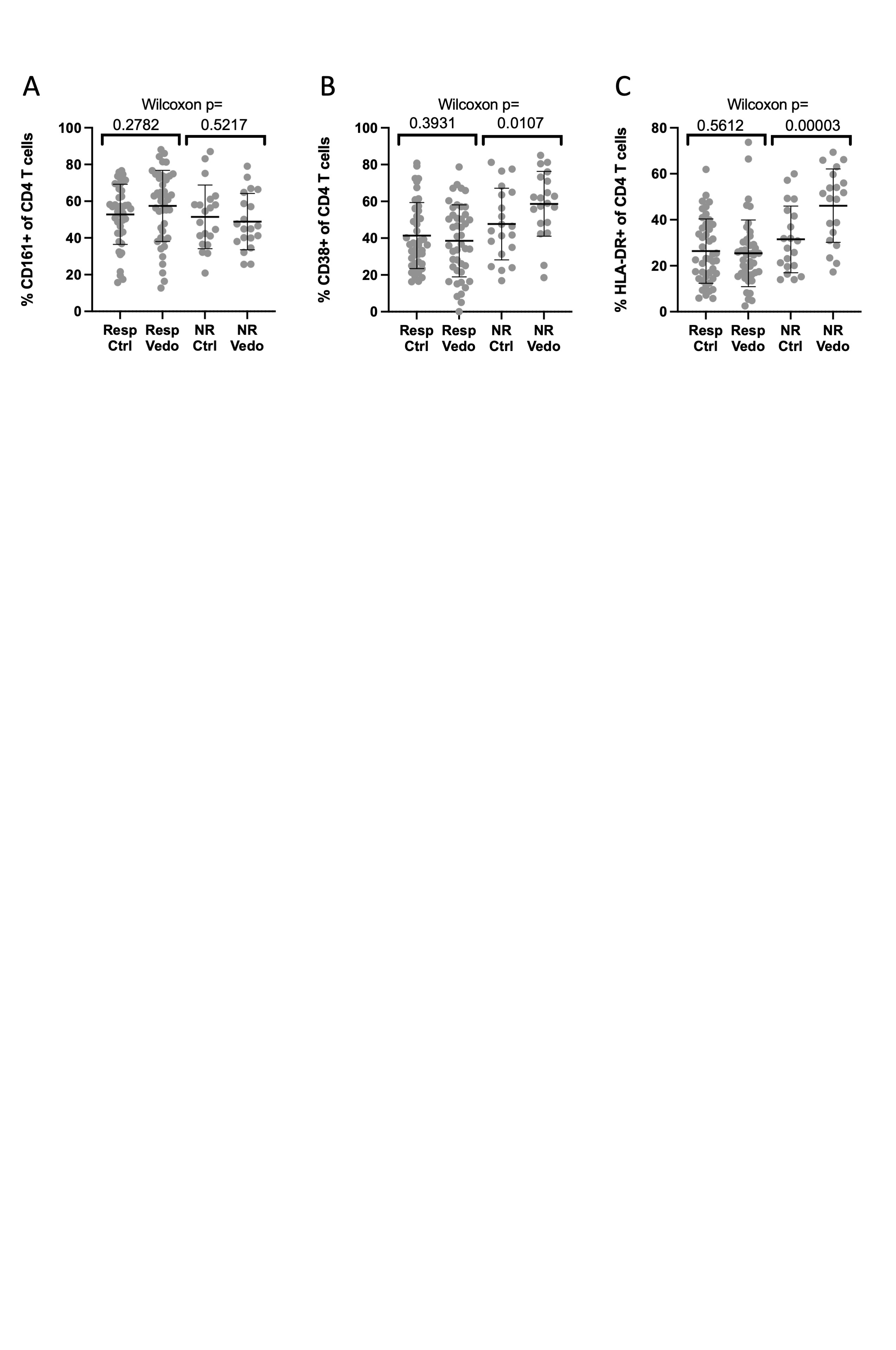

Supplement: izad224_suppl_Supplementary_Figure_S3 [file izad224_suppl_supplementary_figure_s3.jpeg]
