# Supplementary material for: Vedolizumab Efficacy Is Associated With Decreased Intracolonic Dendritic Cells, Not Memory T Cells
Source: Inflamm Bowel Dis. 2023 Oct 14;30(5):704–17. doi: 10.1093/ibd/izad224 (PMC11063563; doi:10.1093/ibd/izad224)
Supplement: izad224_suppl_Supplementary_Figure_Legends [file izad224_suppl_supplementary_figure_legends.docx]

**Supplementary Figure Legends**

**Supplementary Figure 1:** **Concatenated flow cytometry data demonstrating gating strategies** is shown for (**A**) Responders on vedolizumab (**B**) Responder controls (**C**) Nonresponders on vedolizumab (**D**) Nonresponder controls. Black arrows denote nested gating strategy through which data is shown. Specific immune cell populations are labeled. T cell populations sorted for mRNA transcriptome profiling in Figure 2 are denoted with red boxes.

**Supplementary Figure 2: Inflammation in biopsies is confirmed by the relative frequency and phenotype of epithelial cells by flow cytometry.** The frequency of CD326+ (EPCAM+) epithelial cells as a percent of total live cells (**A**), and the percent of such cells expressing HLA-DR (**B**) is plotted for biopsies that were deemed grossly inflamed or uninflamed at the time of endoscopy. CD326 and HLDA-DR expression are shown for biopsies from vedolizumab (Vedo) recipients, separated into treatment-responsive (Resp) and nonresponsive (NR) subsets (**C**, **D**). The above data is re-plotted with vedolizumab recipient data from responders and nonresponders plotted alongside data from their respective controls (**E**, **F**). P-values for unpaired two-way comparisons are shown for data with (Student’s t-test: **A**, **C**) or without (Mann-Whitney test: **B**, **D**) a Gaussian distribution. P-values for paired two-way comparisons between cases and matched controls are shown for data with (paired t-test: **E**) or without (Wilcoxon test: **F**) a Gaussian distribution. Means and standard deviations are shown.

**Supplementary Figure 3: Effect of vedoloizumab on flow cytometric phenotype of CD4 T cells.** The percent of CD4 T cells expressing CD161 (**A**), CD38 (**B**) and HLA-DR (**C**) by flow cytometry in the colon biopsies treatment–responsive (Resp) versus nonresponsive (NR) IBD patients on vedolizumab (Vedo), or their matched controls (Ctrl) is shown. P-values for paired non-parametric two-way (Wilcoxon) comparisons are shown between cases and matched controls. Means and standard deviations are shown.
